# Short-chain fatty acids in patients with schizophrenia and ultra-high risk population

**DOI:** 10.3389/fpsyt.2022.977538

**Published:** 2022-12-12

**Authors:** Huiqing Peng, Lijun Ouyang, David Li, Zongchang Li, Liu Yuan, Lejia Fan, Aijun Liao, Jinguang Li, Yisen Wei, Zihao Yang, Xiaoqian Ma, Xiaogang Chen, Ying He

**Affiliations:** ^1^National Clinical Research Center for Mental Disorders, Department of Psychiatry, The Second Xiangya Hospital of Central South University, Changsha, Hunan, China; ^2^Hunan Key Laboratory of Psychiatry and Mental Health, China National Technology Institute on Mental Disorders, Institute of Mental Health, Hunan Medical Center for Mental Health, The Second Xiangya Hospital of Central South University, Changsha, Hunan, China

**Keywords:** schizophrenia, ultra-high-risk population, short-chain fatty acid, valeric acid, caproic acid

## Abstract

**Background:**

Individuals who experience the prodromal phase of schizophrenia (SCZ), a common and complex psychiatric disorder, are referred to as ultra-high-risk (UHR) individuals. Short-chain fatty acid (SCFA) is imperative in the microbiota-gut-brain axis and brain function. Accumulating amount of evidence shows the connections between psychiatric disorders and SCFAs. This study aims to explore the underlying roles SCFAs play in SCZ by investigating the association of alterations in SCFAs concentrations with common cognitive functions in both the SCZ and UHR populations.

**Methods:**

The study recruited 59 SCZ patients (including 15 participants converted from the UHR group), 51 UHR participants, and 40 healthy controls (HC) within a complete follow-up of 2 years. Results of cognitive functions, which were assessed by utilizing HVLT-R and TMT, and serum concentrations of SCFAs were obtained for all participants and for UHR individuals at the time of their conversion to SCZ.

**Results:**

Fifteen UHR participants converted to SCZ within a 2-year follow-up. Valeric acid concentration levels were lower in both the baseline of UHR individuals whom later converted to SCZ (*p* = 0.046) and SCZ patients (*p* = 0.036) than the HC group. Additionally, there were lower concentrations of caproic acid in the baseline of UHR individuals whom later transitioned to SCZ (*p* = 0.019) and the UHR group (*p* = 0.016) than the HC group. Furthermore, the caproic acid levels in the UHR group are significantly positively correlated with immediate memory (*r* = 0.355, *p* = 0.011) and negatively correlated with TMT-B (*r* = -0.366, *p* = 0.009). Significant differences in levels of acetic acid, butyric acid and isovaleric acid were absent among the three groups and in UHR individuals before and after transition to SCZ.

**Conclusion:**

Our study suggests that alterations in concentrations of SCFAs may be associated with the pathogenesis and the cognitive impairment of schizophrenia. Further researches are warranted to explore this association. The clinical implications of our findings were discussed.

## Introduction

Schizophrenia, a severe and chronic disease with characteristics of delusions, hallucinations, negative symptoms, and cognitive impairment, typically develops in adolescence and early adulthood (1) with its high disability rate causing heavy burden to both society and family (2). Clinical studies have shown that 80–90% patients with schizophrenia experience a prodromal phase of around 3–5 years before the initial onset of the disorder and are referred to as ultra-high risk individuals (3–5). They often present with abnormal emotions, behaviors, and perceptions and 29% of this population will develop SCZ within a 2-year follow-up period (6). Although the pathogenesis of schizophrenia is generally believed to involve a combination of gene-environment interactions (7), many genetic studies have reported that either the genes involved are inconsistent or difficult to replicate (8). As a result, more studies have focused on and emphasized environmental factors (9, 10).

With the concept of microbial-gut-brain axis proposed, the bi-directional connection between the central nervous system (CNS) and gut microbiome has become a major topic in psychiatry. The neural, endocrine, immune, and metabolic pathways are the four widely recognized pathways between the CNS and the gut (11, 12). Dysregulation of gut microbiota has been found in many psychiatric diseases (13–16). Currently, more researches are focusing on the functional transmitters of the gut-brain axis.

Short-chain fatty acids (SCFAs), which mainly consist of acetate acid, propionate acid, butyrate acid, iso-butyric acid, isovaleric acid, valeric acid, and caproic acid, are one of the end metabolites produced by intestinal microbiota (11, 12). They are believed to play an important role in the microbial-gut-brain axis crosstalk. Additionally, SCFAs can enter the systemic circulation and cross the blood-brain barrier (BBB) (17) to provide an imperative regulatory role in the oxidative stress pathways, metabolic pathways, and neurotransmitter pathways, which are all crucial in the biomolecular mechanisms underlying the pathology of schizophrenia and its’ cognitive impairment (18, 19). SCFAs can act as a histone deacetylase inhibitor (HDACI), reinvigorate synaptic plasticity and protect against neuronal damage. The high incidence of schizophrenia in adolescence and early adulthood is thought to be related to several key processes of brain development (20), such as synaptic refinement, myelination, and the physiological maturation of inhibitory neural networks (21–23). Additionally, valproic acid has a SCFA structure (24, 25), act as HDACI, and is one of the most commonly prescribed psychiatric medications (26, 27), although it is not a first-line treatment for schizophrenia, it has a synergistic effect on antipsychotic drugs (28, 29). SCFAs can promote TH1 and TH17 differentiation and induce inflammation (30, 31) while studies have suggested that the onset of schizophrenia is related to the dysfunction of immune and inflammation (32–35). Some studies have shown that mitochondrial dysfunction is associated with the pathogenesis of schizophrenia (36). Butyrate acid is capable of increasing mitochondrial activity, which can help to rectify the disease-associated mitochondrial dysfunction in the brain (37). SCFAs can affect neuroinflammation by regulating microglia, induce the release of a variety of hormones, and regulate the level of neurotrophic factors through histone acetylation, all of which directly or indirectly affect the cognitive function of mental disorders (38). As cognitive impairment such as executive function, attention, memory, and language is intrinsic to schizophrenia, SCFA may have a pivotal effect in SCZ (39, 40).

Although there is no consensus on SCFAs and schizophrenia, researches proved that SCFAs are related to the occurrence of many psychiatric disorders. Patients with anorexia nervosa had lower butyric acid and acetic acid levels than healthy people in addition to lower levels of propionic acid levels after treatment (41). The transplantation of intestinal microbiota from autism disorder (ASD) patients into healthy mice will induce ASD-like behaviors in the animal models (42). Previous studies have furthermore reported that the SCFAs in feces from ASD children are abnormally higher or lower than those of healthy children (43, 44) while in depressed patients, all concentrations of SCFAs are diminished except for the elevated levels of isocaproic acid (45–47).

In the previous study, we assessed the microbial community characteristics of the UHR population (48). The results show that the pathways of pyruvate synthesis, acetyl-CoA synthesis, and fatty acid initial synthesis, which are all components of the synthesis pathways of SCFAs, were revealed to be significantly activated compared with those in the control group. Additionally, we also found higher levels of *Clostridia* in the intestinal tract of the UHR population (14). Concentrations of *Clostridiales* and *Prevotella*, the two main bacterial species responsible for the production of intestinal SCFAs, have been discovered to be increased in the feces of the UHR population. Therefore, we designed this project to explore the role of SCFAs in schizophrenia and its UHR population.

## Materials and methods

### Participants

A total of 59 participants, including 15 participants converted from UHR met the Diagnostic and Statistical Manual of Mental Disorders (DSM-5) criteria for SCZ and never received any medications. After the structured interview for Prodromal syndromes (SIPS), 51 UHR participants with an absence of psychiatric medication history met the diagnostic criteria of Prodromal syndromes (COPS). In total, 40 healthy controls (HC) who did not meet the diagnostic criteria for UHR and SCZ from COPS and DSM-5, respectively, were enlisted based on matching of age, gender, and education levels with individuals from both UHR and SCZ groups. All participants are outpatients recruited from June 2015 to June 2018 at the Second Xiangya Hospital of Central South University in the city of Changsha from Hunan province in China. All participants were 13–30 years old Han Chinese matched with gender and age and gave signed consent for this study. Exclusion criteria for all participants included: gastrointestinal and endocrine diseases (including constipation and diarrhea), serious organ disorders (such as stroke and heart failure), a history of diagnosis of psychiatric disorders and corresponding treatments (such as antipsychotics, antidepressants, and anticonvulsants), had used alcohol, antibiotics, probiotics, or any other medications (oral or injectable) during the last 3 months. The demographic characteristics are detailed in Table 2.

**TABLE 1:** Short-chain fatty acids (SCFAs) regression linear equations.

SCFAs	Linear equation	Coefficient of association (r)	Concentration range (μg/ml)
Acetic acid	y = 0.031x + 0.0173	0.9922	0.02∼100
Propionic acid	y = 0.0512x + 0.0029	0.9963	0.02∼100
Isobutyric acid	y = 0.0806x + 7 × 10^–4^	0.991	0.02∼100
Butyric acid	y = 0.1865x + 0.0055	0.9968	0.02∼100
Isovaleric acid	y = 0.2181x + 7 × 10^–4^	0.9956	0.02∼100
Valeric acid	y = 0.2177x + 0.0012	0.9968	0.02∼100
Hexanoic acid	y = 0.4973x + 0.01	0.993	0.02∼100

**TABLE 2:** Comparison of demographic and clinical characteristics.

Groups	Comparison 1					Comparison 2			
	UHR (*n* = 51)	SCZ (*n* = 59)	HC (*n* = 40)	χ^2^/*F*	*P-*value	UHR-C-B (*n* = 15)	UHR-NC-B (*n* = 36)	χ^2^/*F*	*P-*value
Gender (male/female)	25/26	33/26	24/16	1.153	0.562	8/7	17/19	0.158	0.691
Age (year)	19.00 ± 4.75	20.92 ± 5.59	19.25 ± 2.62	2.337	0.102	20.20 ± 5.51	18.50 ± 4.39	0.504	0.481
Education duration (year)	11.00 ± 2.55	11.10 ± 2.85	12.92 ± 2.56	7.19	<0.001***	10.60 ± 2.59	11.17 ± 2.56	0.006	0.940
SOPS-P score	11.80 ± 5.64	–	–		–	11.80 ± 5.85	11.81 ± 5.63	0.314	0.578
SOPS-N score	11.54 ± 6.51	–	–		–	15.40 ± 5.60	9.94 ± 6.24	0.158	0.693
SOPS-D score	4.02 ± 2.41	–	–		–	4.80 ± 2.60	3.69 ± 2.29	0.170	0.682
SOPS-G score	4.76 ± 3.01	–	–		–	4.87 ± 2.30	4.72 ± 3.29	3.064	0.086
PANSS-P score	–	20.83 ± 7.77							
PANSS-N score	–	25.61 ± 8.24							
PANSS-D score	–	40.49 ± 8.01							
PANSS-S score	–	3.36 ± 11.29							

Comparison 1: UHR, ultra-high risk patients; HC, health controls; SOPS, scale of prodromal symptoms; P, positive symptom; N, negative symptom; D, disorganized symptom; G, general symptom; S, aggressive risk behavior symptoms; Chi-square test was used for gender; Age and years of education were analyzed by one-way ANOVA, at analysis power = 0.8, the effect size *f* = 0.256. Comparison 2: UHR-C-B, the baseline of conversed ultra-high risk patients; UHR-NC-B, the baseline of non-conversed ultra-high risk participants; Chi-square test was used for gender; Age and years of education were analyzed by t-test. ***p < 0.001.

This study was approved by the Ethics Committee of the Second Xiangya Hospital, Central South University (No. 2021YFE0191400) and carried out in accordance with the Declaration of Helsinki. All participants were aware of the risks and benefits of the study and signed informed consent forms.

### Clinical assessment

The Positive and Negative Syndrome Scale (PANSS) was used to evaluate the clinical symptoms of patients with first episode of schizophrenia (FES) at enrollment and include positive, negative, general psychopathological, and aggressive risk behavior symptoms. The Scale of Prodromal Symptoms (SOPS) was used to assess the clinical Symptoms of the clinical high risk (CHR) population at enrollment and includes 19-item scale with four subscales for positive, negative, disorganized and general symptoms. All the participants completed cognitive tests and had their blood drawn. The cognitive tests include portions of MATRICS™ Consensus Cognitive Battery (MCCB™) (49, 50), the Hopkins Verbal Learning Test-Revised (HVLT-R) for analysis of verbal memory, and the Trail Making Test A and B (TMTA, TMTB) for assessment of speed of information processing and standing for executive function. The UHR participants were followed up for 2 years with evaluations on cognitive function, clinical symptoms, and blood samples conducted for individuals whom developed SCZ at the time of transition.

### Serum short-chain fatty acid extraction and estimated

#### Sample preparation

At the time of enrollment, 6 ml peripheral blood was obtained from each participant and centrifuged at 3,000 revolutions per minute (rpm) for 10 min within half an hour. The supernatant, which mostly consist of serum, was stored in a refrigerator at –80°C for further processing. Acetic acid, propionic acid, butyric acid, isobutyric acid, valeric acid, isovaleric acid, and hexanoic acid pure standard quantity was prepared with ether to create twelve mixed standard concentration gradient: 0.02, 0.05, 0.1, 0.2, 0.5, 1, 2, 5, 10, 25, 50, and 100 μg/ml. Both the original mother and working standard solutions were stored at 0°C. A sample of 200 uL were obtained and 100 ul of 15% phosphoric acid were then added. A total of 20 uL of internal standard in the form of 75 ug/ml isohexanoic acid solution and ether were additionally added to homogenate the sample for 1 min. Finally, these were then centrifuged at 12,000 rpm at 4°C for 10 min with the supernatant taken for machine testing. Table 1 is the linear regression equation of each substance, correlation coefficient *r* > 0.99, in a good linear relationship.

#### Gas chromatography-mass spectrometer (GC-MS) analysis

Derivatives were separated by the Agilent HP-InnoWAX capillary column (30 m × 0.25 mmID × 0.25 μm). Helium was used as a carrier gas with a flow rate of 1.0 ml/min. The shunt ratio for shunt injection to injection volume 1 L was 10 to 1. The temperatures of injector, ion source, transmission line and quadrupole were set as 250, 230, 250, and 150°C, respectively. The initial temperature-programmed was 90°C, followed by 10°C/min to 120°C, 5°C/min to 150°C, and finally 25°C/min to 250°C for 2 min. MS conditions: electron bombardment ionization (EI) source, SIM scanning mode, electron energy 70 eV.

#### Fecal DNA extraction and sequencing

Stool samples were collected from 17 participants of the UHR group. DNA were extracted and 16s sequencing were conducted from these samples. Clustering analysis of similar sequences based on valid data revealed a small number of operational taxonomic units (OTU). Species annotation was performed on representative OTU sequences in accordance to the OTU clustering results. The data of relative abundance of fecal flora were thus obtained. For details, see Ref. (48).

### Statistical analysis

Short-chain fatty acids values of zero which were present in more than 30% for each individual SCZ, UHR, and HC groups were not included in the subsequent analysis. Outliers were defined as values outside of the range of mean ± 3 × standard deviation ($\bar{\chi }\pm 3\,\text{SD}$) and were not included in the subsequent analysis. SPSS25.0 statistical software was used to analyze the experimental data. The Chi-square test was used for the categorization of gender variables and the results were represented by proportion (male/female). One-way ANOVA was used for age and years of education in accordance with normal distribution, and the results were represented by mean ± standard deviation ($\bar{\chi }\pm \text{s}$). Cognitive function was analyzed by co-variance analysis, years of education and age were included as co-variable. The non-normal distribution of acetic acid, butyric acid, iso-valeric acid, valeric acid, and caproic acid among the three groups were tested by the Kruskal–Wallis test with the results expressed as median (upper quartile, lower quartile) [M(Q_L_,Q_U_)]. Variance analysis was used for the normal distribution of the total and the results were expressed as mean ± standard deviation ($\bar{\chi }\pm \text{s}$). Spearman correlation analysis was used to analyze the correlation between SCFAs level and cognitive function and clinical symptoms. Spearman rank correlation was also used to analyze the correlation between SCFAs and the relative abundance of gut microbiota. Bilateral *p* < 0.05 indicates a statistical difference, the results were corrected by Bonferroni. *Post-hoc* tests were performed for all significant *P*-values and effect size were calculated for an analysis power = 0.8 by using the software G*power and *Psychometrica.*

## Results

### Demographic and clinical characteristics

The demographic and clinical characteristics are showed in Table 2. There were no significant differences in gender (*p* = 0.562) and age (*p* = 0.102) among the three groups but SCZ and UHR participants had significant shorter education duration than HC participants (*p* < 0.001), and consider the young age of UHR population, we adopted education duration and age as the covariables in further analysis. During our 2-year follow-up, 15 of the 51 UHR participants developed SCZ with a conversion rate of around 29.41%. As this rate is consistent with the reports from the Shanghai SHARP project and previous international studies (51), we compared the demographic characteristics and clinical information of the converted and non-converted population in the UHR group at baseline levels. There were no significant differences in gender (*p* = 0.562), age (*p* = 0.102), education duration (*p* = 0.940), and SOPS scores (*p*sops-P = 0.578, *p*sops-N = 0.693, *p*sops-D = 0.682, *p*sops-G = 0.682).

We found that the cognitive functions of the UHR group and the SCZ group were significantly impaired when compared with that of the HC group while some cognitive functions of the SCZ group were further diminished than that of the UHR group (Table 3). The specific performance is as follows: The immediate recall (*p* = 0.028, *p* < 0.001) of the UHR group and the SCZ group were worse than that of the HC group in the HVLT-R test. The delayed recall in the SCZ group was also worse than that in the HC group (*p* < 0.001). Compared with the UHR group, the SCZ group performed worse in delayed recall (*p* = 0.022) for the HVLT-R test. There was no significant difference between the UHR group and the HC group for finishing the TMT-A and TMT-B tests, but the SCZ group took significantly longer than the HC group (*p* = 0.007, *p* = 0.014). At the same time, we also compared and analyzed the cognitive function of the UHR-C-B group and the UHR-NC-B group and observed no significant differences between the two group (Table 3).

**TABLE 3:** Comparison of cognitive domains.

Cognitive domains	Comparison 1						Comparison 2			
	UHR (51)	SCZ (58)	HC (40)	*F*	*p*	*η^2^*	UHR-C-B (15)	UHR-NC-B (15)	*F*	*p*
**HVLT-R**										
Immediate recall	5.76 ± 0.30	4.94 ± 0.28	7.04 ± 0.34	8.324	<0.001***	0.104	5.07 ± 1.44	5.94 ± 2.40	2.866	0.097
Delayed recall	8.84 ± 0.38	7.21 ± 0.35	10.12 ± 0.44	10.28	<0.001***	0.125	8.40 ± 2.32	8.89 ± 2.97	0.859	0.358
**TMT completion time**										
TMT-A	42.33 ± 3.65	52.77 ± 3.42	33.91 ± 4.24	4.987	0.008**	0.065	43.62 ± 13.67	42.75 ± 15.89	0.063	0.802
TMT-B	112.91 ± 9.57	136.56 ± 8.96	90.01 ± 11.10	4.20	0.017*	0.055	114.48 ± 53.93	118.37 ± 86.52	1.557	0.218

HVLT-R, the hopkins verbal learning test-revised; TMT, trail making test. Education duration and age were taken as covariable and covariance-analysis was used. *p < 0.05, **p < 0.01, ***p < 0.001.

### Comparison of short-chain fatty acids level between different groups

Subsequent analysis were not conducted out for propionic acid and isobutyric acid because the 0 value of each in the SCZ, UHR, and HC groups exceeded 30% (propionic acid values of 44.07, 45.10, and 52.50% and isobutyric acid values of 42.37, 35.29, and 35%, respectively). By using the criteria of ($\bar{\chi }\pm 3\,\text{s}$), the following outliers were identified and removed: the data with an acetic acid content of 5.778 and the data with a caproic acid content of 0.146 in the SCZ group; the data with a caproic acid content of 0.347 in the UHR group; the data with an acetic acid content of 5.998, and the data with an iso-valeric acid content of 0.139 in the HC group. In addition, the total amount of SCFA corresponding to the forementioned data was not calculated for the samples in Table 4.

**TABLE 4:** Comparison of short-chain fatty acid (SCFA) level between different groups.

SCFA (μg/ml)	Comparison 1						Comparison 2					
	UHR	SCZ	HC	H/F	*p*	d_Cohen_	UHR-C-B	UHR-NC-B	HC	H/F	*p*	d_Cohen_
Acetic acid	1.87 (1.38, 2.72)	1.93 (1.38, 2.91)	2.14 ± 1.08	0.31	0.852		2.03 ± 1.18	1.84 (1.44, 2.77)	2.14 ± 1.08	0.319	0.890	
Butyric acid	0.05 (0.03, 0.08)	0.05 (0.03, 0.08)	0.07 ± 0.03	3.82	0.148		0.04 (0.03, 0.08)	0.06 (0.03, 0.09)	0.07 ± 0.03	3.40	0.182	
Isovaleric acid	0.04 (0.02, 0.06)	0.04 (0.00, 0.05)	0.04 (0.03, 0.06)	3.20	0.202		0.04 ± 0.02	0.04 (0.02, 0.08)	0.04 (0.03, 0.06)	1.086	0.581	
valeric acid	0.03 (0.00, 0.07)	0.00 (0.00, 0.06)	0.04 ± 0.03	6.35	0.042*	0.349	0.00 (0.00,0.03)	0.03 (0.00, 0.07)	0.04 ± 0.03	6.01	0.049*	0.437
caproic acid	0.03 (0.00, 0.05)	0.05 (0.00, 0.06)	0.05 (0.04, 0.08)	8.17	0.017*	0.422	0.00 (0.00, 0.05)	0.04 (0.00, 0.05)	0.05 (0.04, 0.08)	9.067	0.011*	0.595
The total	2.23 ± 1.00	2.39 ± 1.14	2.32 ± 1.09	0.26	0.769		2.01 ± 1.09	2.32 ± 0.96	2.32 ± 1.09	0.105	0.901	

Comparison 1: Acetic acid, butyric acid, isovaleric acid, valeric acid, and caproic acid were tested by the Kruskal–Wallis. One-way ANOVA was used for the total. Adopted Bonferroni correction. Acetic acid (UHR = 51, SCZ = 58, HC = 39), butyric acid (UHR = 51, SCZ = 59, HC = 40), iso-valeric acid (UHR = 51, SCZ = 59, HC = 39), valeric acid (UHR = 51, SCZ = 59, HC = 40), Caproic acid (UHR = 50, SCZ = 58, HC = 40), the total (UHR = 50, SCZ = 57, HC = 38). Comparison 2: UHR-C-B, the baseline of conversed ultra-high risk patients; UHR-NC-B, the baseline of non-conversed ultra-high risk participants; Acetic acid, butyric acid, iso-valeric acid, valeric acid, and caproic acid were tested by Kruskal–Wallis. One-way ANOVA was used for total quantity. *p < 0.05; Bonferroni correction was used. Acetic acid (UHR-C-B = 15, UHR-NC-B = 36, HC = 39), butyric acid (UHR-C-B = 15, UHR-NC-B = 36, HC = 40), iso-valeric acid (UHR-C-B = 15, UHR-NC-B = 36, HC = 39), valeric acid (UHR-C-B = 15, UHR-NC-B = 36, HC = 40), caproic acid (UHR-C-B = 14, UHR-NC-B = 36, HC = 40), the total (UHR-C-B = 14, UHR-NC-B = 36, HC = 38). *p < 0.05.

**FIGURE 1:**
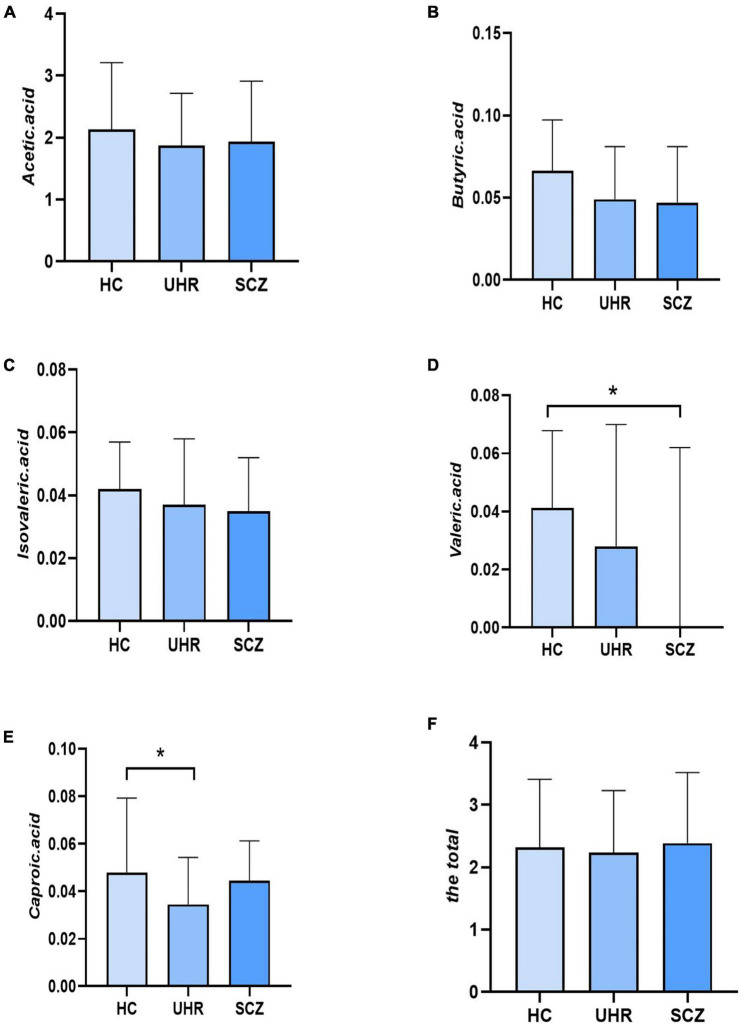
The short-chain fatty acids (SCFAs) level comparison in healthy controls (HC) group, ultra-high-risk (UHR) group, and schizophrenia (SCZ) group. **(A–F)** Showed the comparison of acetic acid, butyric acid, isovaleric acid, valeric acid, caproic acid and the total SCFAs levels, respectively among Group HC, Group UHR and Group SCZ. *p < 0.05.

**FIGURE 2:**
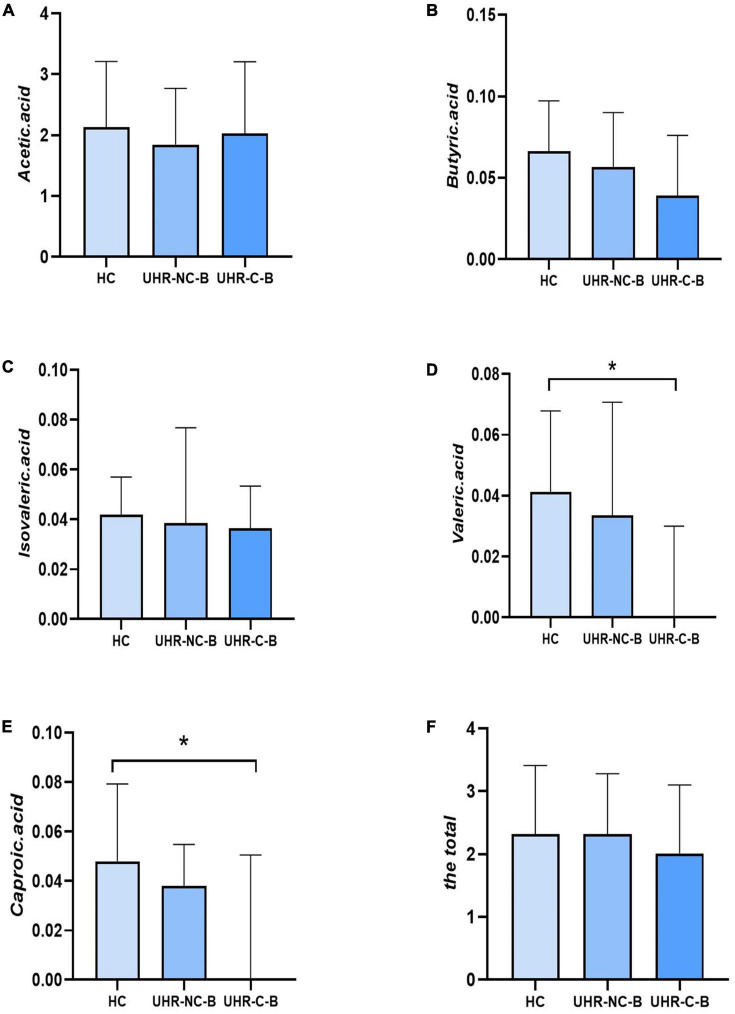
The comparison of short-chain fatty acids (SCFAs) content among the baseline of converted ultra-high risk participants (UHR-C-B), the baseline of non-conversed ultra-high risk participants (UHR-NC-B), and the healthy controls (HC) group. **(A–F)** Showed the comparison of acetic acid, butyric acid, isovaleric acid, valeric acid, caproic acid and the total SCFAs levels, respectively among Group HC, Group UHR-NC-B and Group UHR-C-B. *p < 0.05.

Table 4 and Figure 1 showed the SCFAs level in UHR group, SCZ group and HC group. There were no significant differences in acetic acid, butyric acid, isovaleric acid, and total SCFAs’ level in the SCZ group and the baseline of the UHR group and the HC group. The level of valeric acid in the SCZ group was significantly lower than that in the HC group (*p* = 0.036) while the content of caproic acid in the UHR group was significantly lower than that in the HC group at baseline (*p* = 0.016). Table 4 and Figure 2 showed the comparison of SCFAs content among the baseline of converted ultra-high risk participants (UHR-C-B), the baseline of non-conversed ultra-high risk participants (UHR-NC-B) and the HC group. The contents of valeric acid (*p* = 0.046) and caproic acid (*p* = 0.019) in the UHR-C-B group at baseline were significantly lower than those in the HC group, while there were no significant differences in acetic acid, butyric acid, isovaleric acid, and total SCFA level among the UHR-C-B, UHR-NC-B, and HC groups. Finally, in our study, 15 of the 51 ultra-high-risk patients developed schizophrenia during the 2 years follow-up with a conversion rate of about 29.41%, which was consistent with previous studies on the UHR population. There was no other significant difference in the content and total amount of SCFAs in these participants before and after conversion.

### Correlation analysis of short-chain fatty acids with cognitive function and gut microbiota

As valeric acid in the SCZ group and caproic acid in the UHR group were significantly reduced, these two SCFA were compared with the different significant alterations in cognitive function (SCZ group: immediate recall, delayed recall, TMTA, TMTB; UHRZ group: immediate recall) with the results of the analysis are presented in Table 5. We found that valeric acid in SCZ patients were not correlated with HVLT-R and TMT test, while caproic acid levels in UHR patients were significantly positively correlated with immediate memory (*r* = 0.355, *p* = 0.011) and negatively correlated with TMT-B (*r* = -0.366, *p* = 0.009).

**TABLE 5:** Correlation analysis of short-chain fatty acids (SCFAs) with cognitive function.

Groups	HVLT-R		TMT	
	Immediate recall	Delayed recall	TMT-A finish time	TMT-B finish time
**SCZ-valeric**				
r	–0.140	–0.125	0.085	0.228
*p*	0.291	0.345	0.522	0.088
**UHR-caproic**				
r	0.355	0.302	–0.240	–0.366
*p*	0.011*	0.033	0.093	0.009*

Spearman correlation analysis and Bonferroni correction was used, *p < 0.0125 (0.05/4) have a significant difference. At the analysis power = 0.8, the effect size = 0.375.

**FIGURE 3:**
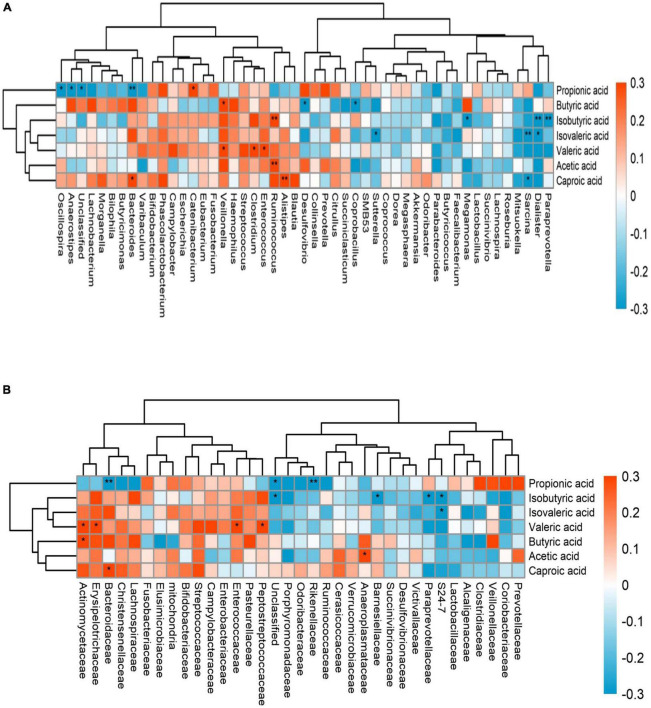
The abscissa is the name of gut microbiota, and the ordinate is the name of short-chain fatty acids (SCFAs). Each square crossed by abscissa and ordinate represents the correlation and significance of the two. The size of the correlation coefficient is expressed by the difference and depth of color, red represents positive correlation, blue represents negative correlation, darker color, higher correlation, lighter color, lower correlation; *: 0.01 < *p* < 0.05; **: 0.001 < *p* < 0.01. **(A)** showed the correlation between serum SCFAs and fecal flora at genus level, **(B)** showed the correlation between serum SCFAs and fecal flora at family level.

**FIGURE 4:**
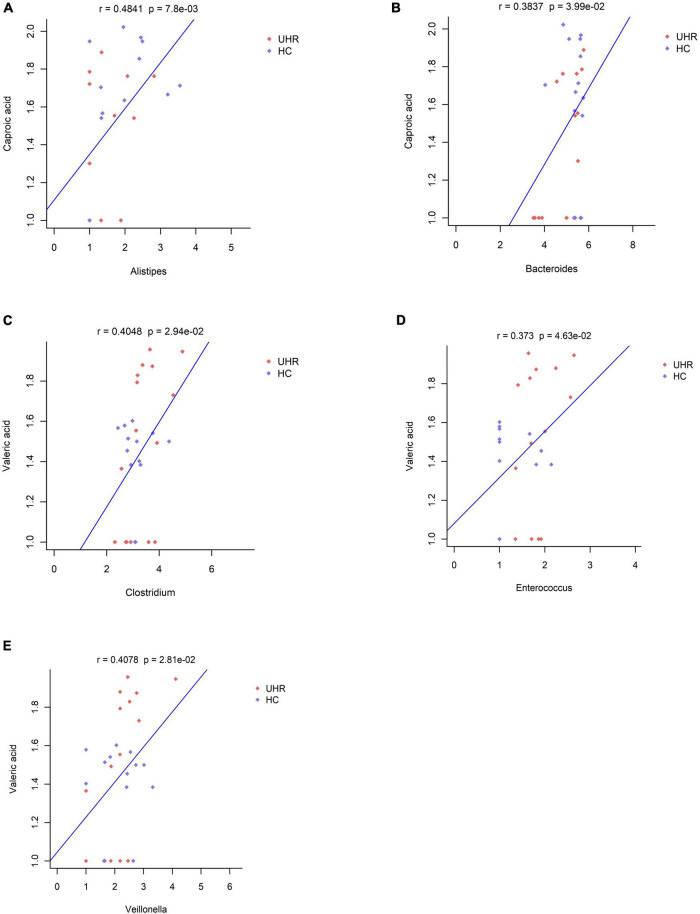
Correlation between serum short-chain fatty acids (SCFAs) and the abundance of fecal *Enterococcus*, *Clostridium*, *Veillonella*, *Alistipes*, and *Bacteroides* genus level. **(A)** Showed the correlation between caproic acid level and the abundance of Alistipes. **(B)** Showed the correlation between caproic acid level and the abundance of *Bacteroides*. **(C–E)** Showed the correlation between valeric acid level and the abundance of *Clostridium*, *Enterococcus*, and *Veillonella*, respectively.

**FIGURE 5:**
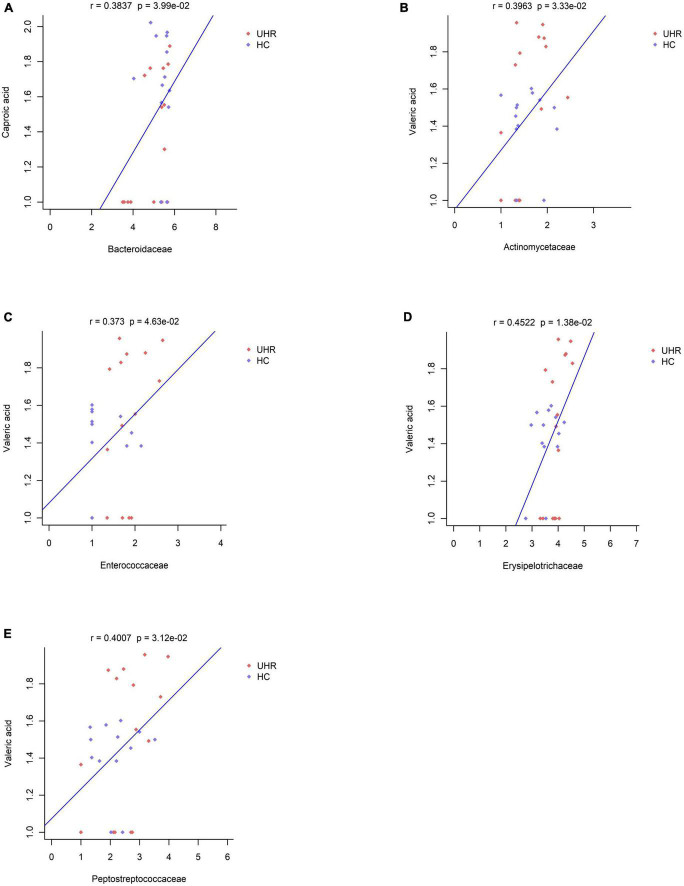
Correlation between serum short-chain fatty acids (SCFAs) and the abundance of fecal *Peptostreptococcaceae*, *Enterococcaceae*, *Erysipelotrichaceae*, *Actinomycetaceae*, *Bacteroidaceae* at family level. **(A)** Showed the correlation between caproic acid level and the abundance of *Bacteroidaceae*. **(B–E)** Showed the correlation between valeric acid level and the abundance of *Actinomycetaceae*, *Enterococcaceae*, *Erysipelotrichaceae*, and *Peptostreptococcaceae*, respectively.

We combined the relative abundance of fecal intestinal flora in the UHR population, and conducted correlation analysis with serum SCFAs. For valeric acid and caproic acid, the results were shown at the genus level (Figure 3A). The content of valeric acid in the patients’ serum was positively correlated with the relative abundance of *Enterococcus*, *Clostridium*, *Veillonella*, whereas caproic acid was positively correlated with the relative abundance of *Alistipes* and *Bacteroides*. At the family level (Figure 3B), the content of valeric acid in serum was positively correlated with the relative abundance of *Peptostreptococcaceae*, *Enterococcaceae*, *Erysipelotrichaceae*, and *Actinomycetaceae*, while caproic acid was positively correlated with the relative abundance of *Bacteroidaceae*. Figures 4, 5 shows the correlation scatter diagram.

## Discussion

### Cognitive function analysis of schizophrenia patients and its ultra-high-risk population

In our study, the immediate memory of the UHR group was significantly impaired when compared with that of the HC group. Additionally, both memory function and delayed memory of the SCZ group was significantly more impaired than those of the UHR group. These results suggest that the UHR population has memory impairment that is similar but milder than SCZ patients. Earlier studies provided consistent results by indicating presence of widespread non-selective memory impairment in patients with SCZ (52, 53) whereas the memory function of UHR individuals has a significant trend of initial impairment (54) though the exact specific type of memory impairment was not clarified. Studies associated with the memory related cortical-limbic system have indicated extensive functional impairment in SCZ patients (55, 56), whereas only structural abnormalities with an absence of functional abnormalities were detected in the genetic high risk population (57). However, larger sample size of the UHR population are required for further in-depth exploration and analysis. In terms of memory mechanism, short-term memory is mainly related to the enhancement of synaptic connections, while long-term memory is related to the establishment of new synapses. Dysregulation of these synaptic function is an important pathophysiology of schizophrenia (21–23). Immediate memory provides time for us to judge the importance of peripheral stimuli and the formation of long-term memory is dependent on its continuous repetition. As a result, the decline of immediate memory in UHR may potentially affect long-term memory in SCZ patients to some extent. Therefore, we speculate that immediate memory impairment due to partial memory damage is one of the prodrome of schizophrenia and injury of the delayed memory induced by comprehensive impairment of memory function may be a sign of the formal onset of SCZ.

The TMT completion time reflects information processing speed and represents one of the executive functions. In our study, completion time were significantly longer in the SCZ patients for the TMT test. Furthermore, although the average completion time was longer in the UHR group than in the HC group, there were no significant level of difference. This is consistent with the core negative symptom of reduced volitional activity in SCZ patients as SCZ patients present with a decline in executive function and difficulty completing previously familiar learning and work events (58–60). Additional study has discovered that the defects of execution speed were one of the most sensitive indicators of predictive transformation (61).

In conclusion, consistent with previous studies, UHR patients do have similar but less severe cognitive impairment including memory and executive function than SCZ patients (62–65).

### Short-chain fatty acids level analysis of schizophrenia patients and its ultra-high-risk population

Our research is the first longitudinal study to investigate the serum short-chain fatty acid concentration levels in UHR individuals and SCZ patients with a 2-year follow-up of the UHR sample group. We discovered serum valeric acid levels in the SCZ group and caproic acid levels in the UHR group were lower than those in the HC group. Additionally, during the follow-up, the levels of valeric acid and caproic acid in the initial baseline of UHR individuals who converted to SCZ were significantly lower than those in the HC group.

Previous studies about SCFAs levels in serum or feces of SCZ patients have been controversial and focused more on acetic acid, propionic acid, and butyric acid, often ignoring SCFAs with low content such as valeric acid and caproic acid. For example, Huihui et al. found that the levels of acetic acid, isovaleric acid and hexanoic acid in feces of SCZ patients increased while the levels of isobutyric acid decreased (66). Although there was no change in serum butyric acid level in SCZ patients, it increased after treatment and was positively correlated with the treatment effect (67). Due to intestinal absorption regulation and liver metabolism (38), only a small fraction of SCFAs can enter the circulation. Lai et al. also found that the concentration of SCFAs in feces was much higher than that in blood (68), which partly explains the fact that propionic acid and isobutyric acid was undetected in more than 30% of our samples. Few results from other studies are consistent with our findings and may be related to our selection of serum as samples and the inclusion of UHR population for analysis. However, as serum is closer to the BBB than feces, it is still a better choice than feces.

In our study, serum valeric acid levels in the SCZ group and in the baseline of UHR individuals who converted were significantly lower than those in the HC group. Most studies suggest that valeric acid in the body is beneficial. Valeric acid structurally resembles GABA, and has been shown to bind the GABA a receptor (69) while GABA deficiency is one of the important pathological hypotheses in schizophrenia. Valeric acid is a HDACI (69, 70) and *in vivo* study found that patients with schizophrenia have histone deacetylases dysregulation (71). Valeric acid also exerts anti-inflammatory effects by reducing NF-KB transactivation (72, 73) while neuroinflammation is present in both SCZ patients and UHR people (74–77). Previous study also proved that valeric acid can promote the maturation of neurons (73) as alterations of valeric acid level may directly affect neurodevelopment and may be thus associated with the high incidence of schizophrenia in adolescence and early adulthood (21–23). These findings, which suggest that decrease of valeric acid may be related to the decreased anti-inflammatory ability of the body and can potentially lead to disorder of neural maturation, are consistent with our findings. On the contrary, one study has found that increased serum valeric acid levels lead to inflammatory responses and can damage the formation and dendritic structures of brain cells in mice (68). This contradictory result may be due to difference in biological samples as our study included human samples and valeric acid may potentially be more advantageous for mice. Our study shows that both SCZ patients and UHR individuals during the prodromal phase who later converted to SCZ had reduced valeric acid levels. Based on the inflammatory hypothesis and neurodevelopmental hypothesis, we postulate that valeric acid plays a protective role *in vivo* and decreased level of valeric acid may affect the anti-inflammatory and neuronal maturation abilities. We thus hypothesize that valeric acid may be a predictor of transformation in the UHR population and may be associated with the onset of SCZ. However, this theory needs to be explored with a larger sample size in the future.

In the current study, the level of caproic acid in the UHR population and in both the UHR group and the baseline of UHR individuals who developed into SCZ are lower than those of the HC group. We also found that caproic acid may affect cognitive function more as the impaired memory of the UHR group is related to decrease of serum caproic acid level and the lower the level of caproic acid, the worse the memory function become. It is worth mentioning that though there was no significant difference in TMT test performance between the UHR and HC group, there was a significant negative correlation between level of caproic acid and the completion time of TMT-B. This probably indicates that alterations in caproic acid is more sensitive than commonly used performing test. Consistent with our study, caproic acid may works similarly to valeric acid in patients with SCZ by providing a protective purpose in the human body as a participant of anti-IL-32 and anti-inflammatory effect (78). In animal and plants, caproic acid can maintaining microbial homeostasis, preventing expansion of pathogenic bacteria (79–81). Therefore, although caproic acid is toxic in industry, trace amount of caproic acid in the human body is potentially beneficial and may play a protective role in the human body. In future research, more evidence is needed to show whether and how caproic acid is involved in the process of cognitive impairment in the UHR and SCZ population.

In recent years, inflammation and immunity have been gradually proven to play a crucial role in the pathogenesis of SCZ. A large number of SCZ patients have persistent chronic inflammation with alterations in levels of cytokine and C-reactive protein (CRP). Significant increase in concentrations of IL-1, IL-6, IL-8, and other cytokines have not only been consistently detected in the serum (82, 83), but also in the cerebrospinal fluid (CSF) (84, 85). Our results support this as in our study, both valeric acid and caproic acid demonstrate anti-inflammatory effects. As these concentrations were lowered in SCZ patients and UHR individuals, their anti-inflammatory abilities were diminished which may closely be related to the induction and aggravation of neuroinflammation.

Although valeric acid and caproic acid have different unique mechanisms to protect the human body, they also have many common functions. Valeric acid and caproic acid both have anti-inflammatory effects and can pass the blood-brain barrier (BBB) (86, 87), allowing them to affect the central nervous system (CNS) directly. Additionally, it is imperative to note that valeric acid and caproic acid both are analogs of valproic acid (88) which is a classic mood stabilizer often used as adjunctive medication in patients with schizophrenia (89). Valeric acid and caproic acid may thus play a similar role like valproic acid in promoting neuroprotection and neurodevelopment, providing a protective and beneficial effect for the human body. As epidemiological evidence on the relationship between valeric acid, caproic acid and schizophrenia is limited, we need to find more direct evidence to explain the underlying mechanism of these SCFAs behind the pathogenesis of schizophrenia and the relationship with cognitive impairment.

The valeric acid in the serum was positively correlated with the relative abundance of *Enterococcus*, *Clostridium*, *Veillonell*, *Peptostreptococcaceae*, *Erysipelotrichaceae*, and *Actinomycetaceae* in the fecal while caproic acid was positively correlated with the relative abundance of *Alistipes* and *Bacteroides*. Previous studies showed that all of the *Alistipes*, *Bacteroides*, *Enterococcus*, *Clostridium*, *Veillonella*, *Peptostreptococcaceae*, *Erysipelotrichaceae* can produce SCFA (90–92). It is worth mentioning that studies have shown that *Alistipes*, *Bacteroide*, and *Clostridium* are closely related to diseases of the mental system. *Alistipes* can affect tryptophan and is related to GABA (93) which is closely concerned with the pathogenesis of schizophrenia. The level of *Alistipes* and *Bacteroides* are increased in depressed patients (69) and decreased in patients with autism (69, 94, 95). Meanwhile, *Bacteroides* are related to neural development as studies have shown that infants with high *Bacteroides* content in their intestine at the age of 1 year have better cognitive and language development after 1 year, especially boys (96). *Clostridium* produces endotoxins in the gut that promote inflammation (97). Our previous study showed that the relative abundance of *Clostridium* increased in SCZ patients, and we speculated through bioinformatics analysis that intestinal SCFA production increased in UHR patients. Combined with the direct measurement results of serum SCFA, serum valeric acid and caproic acid contents decreased, and due to data limitations, we lacked the analysis of total SCFA content. This may be related to the inconsistent changes in SCFA levels in the intestinal tract and SCFA levels in feces and serum in the UHR population. Both flora and SCFA are complex combinations. Maybe only by finding out the effects of specific flora and SCFA on diseases can we treat and intervene in diseases more accurately. This is also the direction of further research in the future.

### Limitations

The main limitations of our study are as follows. First, our sample size was not large enough, including the number of UHR individuals whom converted to SCZ later on. Thus, the comparison results before and after conversion may not be accurate enough. Second, the number of stool samples is too small. If sufficient stool samples were included for association analysis, the results would have been more reliable and accurate. Thirdly, as it is impossible to determine directly the level of SCFA in the CSF, we selected serum as samples because serum is closer to CSF in comparison to feces. However, as most of the SCFAs are metabolized, some SCFAs did not reach a measured value of more than 30% and were excluded in order to not affect the analysis results. Finally, we lack some important factors related to SCFAs, such as Body Mass Index (BMI), personal eating habits, and intestinal microbial condition, were not measured. Although the eating habits in Hunan province are very similar, we should control for these factors in future studies to reduce possible bias.

## Conclusion

In this study, we combined cross-sectional and longitudinal analysis. Up to our current knowledge, we are the first to explore changes in serum SCFAs levels, analyze the relationship between alterations in SCFAs concentration and impaired cognitive function, and examine the correlation between SCFAs and intestinal microbiota. We reported new findings that indicate lower levels of valeric acid were present in both UHR individuals before conversion and patients with SCZ. Additionally, in the UHR population, levels of caproic acid were significantly reduced and is correlated with memory and executive functions. Our results suggest that valeric acid and caproic acid may be involved in the conversion of the UHR population, more studies with large sample sizes need to be explored in the future, and hope to provide further potential knowledge of the pathophysiological mechanisms underlying SCZ.

## Data availability statement

The raw data supporting the conclusions of this article will be made available by the authors, without undue reservation.

## Ethics statement

The studies involving human participants were reviewed and approved by the Ethics Committee of the Second Xiangya Hospital, Central South University (No. 2021YFE0191400). Written informed consent to participate in this study was provided by the participants’ legal guardian/next of kin.

## Author contributions

HP, YH, and XC designed the study. LO, LY, LF, AL, JL, YW, ZY, XM, and ZL collected the samples and clinical information. HP, YH, ZL, and XC analyzed and discussed the experimental result. HP and DL wrote the first draft of the manuscript. All authors contributed to the article and approved the final manuscript.
